# Three-dimensional geometry of human tibial anterior curvature in chronologically distinct population samples of Central Europeans (2900 BC – 21^st^ century AD)

**DOI:** 10.1038/s41598-019-40625-3

**Published:** 2019-03-12

**Authors:** Hana Brzobohatá, Václav Krajíček, Petr Velemínský, Jana Velemínská

**Affiliations:** 1 Department of Prehistorical Archaeology, Institute of Archaeology of the Academy of Sciences, Prague, Czech Republic; 2 Department of Anthropology and Human Genetics, Faculty of Science, Charles University, Prague, Czech Republic; 3 Department of Anthropology, National Museum, Prague, Czech Republic

## Abstract

Several lines of bioarchaeological research have confirmed the gradual decline in lower limb loading among past human populations, beginning with the transition to agriculture. The goal of this study was to assess whether human tibial curvature reflects this decline, with a special emphasis on the time-span during which the pace of technological change has been the most rapid. Our study is the first (1) to apply longitudinal curvature analysis in the antero-posterior (A–P) and medio-lateral (M–L) planes to the human tibia, and (2) that incorporates a broad temporal population sample including the periods of intensification of agriculture, urbanization and industrialization (from 2900 BC to the 21^st^ century AD; N = 435) within Czech territories. Using three-dimensional geometric morphometrics, we investigated whether anterior tibial curvature mirrors assumed diminishing lower limb loading between prehistoric and industrialized societies and explored its shape in all three dimensions. Results showed the continuous trend of A–P straightening of the shaft. This straightening was associated with a relative sigmoidal curve accentuation in the M-L plane. Given the timescale involved and the known phenomenon of declining mobility, such adaptive changes in bone geometry can be interpreted in terms of the diminishing biomechanical demands on the tibia under different living conditions.

## Introduction

Because long-bone curvature develops in only a normal developmental context, it is natural to assume that this variable conveys some functional advantage to the bone or to its immediate biological environment. It should be apparent that regarding the mechanical consequences of bone shaft curvature, any biomechanical role is difficult to assess and may well vary markedly across limb segments and species for any given limb segment^1^. This feature has an apparent relationship with both activity level and locomotion in humans^1–3^ and non-human primates^4^, while its ontogenetic development depends on normal muscle activity as well as weight-bearing^5^.

Most long bones are curved along their axis, a morphological characteristic that augments rather than reduces the mechanical strain caused by bending^6^. In bones involved in locomotion, curvature is a complex feature to quantify, and its biomechanical environment is difficult to model, because it is subject to different strains during the gait cycle^5,7^. Functional explanations for the existence of bending are largely theoretical, however, and are subject to debate^8–12^. Curvature has possibly evolved to lower bending stress by translating it into axial compression^8^ Stress reduction may not be the only advantage of bone curvature, because it may also facilitate muscle expansion and packing^9^, or generate strains necessary for optimal bone strength^5^ or make the manner in which a bone bends more predictable^6^.

Very few studies to date have addressed variation in longitudinal curvature of limb bones within humans^1–3,13^. Shackelford and Trinkaus^1^ measured A-P femoral curvature as distance from longitudinal chord and documented temporal decline in its degree that accompanied declining mobility levels from Neanderthals through early modern to recent humans. The more up-to-date but traditional osteometric work of Macintosh *et al*.^3^ assessed A-P tibial curvature and its relationship with diaphyseal cross-sectional geometry and body size in preindustrial Europeans spanning 6,150 years following the introduction of agriculture. The results of this study support a relationship between tibial curvature and cross-sectional geometry and suggest that changes in mechanical loading may have influenced a suite of morphological features related to bone adaptation in the lower limb. Declining tibial A-P curvature was accompanied by a simultaneous temporal decline in cross-sectional geometric properties, producing a pattern of declining robusticity and curvature through time.

De Groote^2^ used semi-landmark methods to explain variation in longitudinal bone curvature in the femur, radius and ulna amongst human populations from the present-day and Holocene who were geographically, temporally and behaviourally diverse. This study revealed that femoral A–P curvature is related to habitual activity patterns and is unrelated to climate; the greatest curvature levels are therefore found in the samples with the presumed highest activity levels. Although, in different size mammals, long bone curvature is known to scale positively with body weight^14^, in humans, there was found to be no correlation between body size and bone curvature^2^.

In this analysis, we investigated tibial shape by using 3D morphometric semi-landmark methods to identify and quantify phenotypic variation in anterior crest curvature within a chronologically diverse sample of pre-historic, historic, recent and modern human adult bones. The primary goal of this study was to assess whether, and to what extent, tibial anterior crest curvature reflects the shifts associated with changes in subsistence and technological advances among a temporally and behaviourally varied range of populations. Archaeological skeletal collections are limited because they can usually be used only to make broad cultural generalizations about habitual behaviour and mobility. Mobility is usually broadly defined as the habitual amount of traveling (either through walking or running) that characterised a population. Prehistoric and historic samples are usually expected to reflect a mixture of the two types of mobility: residential (i.e. movement of a residential settlement from one location to another) and logistical (foraging movements of individuals or small groups from and back to the base residential site)^15^. Leaving aside this classification, the general stressfulness of the mechanical environment of the samples under study can vary depending on the type/intensity of agriculture, water availability, available technologies, urbanization and industrialization.

In the five pre-industrial Central European samples in this study, the Late Eneolithic Corded Ware (between 2900 BC and 2500 BC) and Bell Beaker datasets (between 2500 BC and 2200 BC) are the chronologically oldest^16^. On the basis of archaeological records, including almost negligible evidence of sedentary agricultural activities and settlement features, it has been suggested that these groups were highly mobile and that their subsistence strategies depended heavily on herding and seasonal movements among pasture zones and temporary settlements. The question of the degree of mobility of the Central European Eneolithic groups has recently been solved using strontium isotope analysis, with results indicating substantial mobility in this period^17^. However, recent biomechanical analyses of the tibial and femoral shaft do not support an extremely high degree of mobility in the Late Eneolithic Central Europe. Cross-sections and overall mobility differences between the Late Eneolithic and Early Bronze Age periods were minute and the changes in mobility between the two periods were not unidirectional, i.e. diachronic^18,19^. No matter what the residency status of these Eneolithic populations was, we can expect that they lived the most physically demanding lifestyle due to the fact that they were less technologically advanced than the comparison groups.

Later, after 2500 BC, daily practices would have changed, thus leaving behind more visible traces in the archaeological record. The lifestyles of Early Bronze Age populations (between 2300 BC and 1600 BC) are thought to have been sedentary; these people relied on agricultural means of subsistence, with an unclear proportion of plant cultivation and animal breeding. Even though the advent of bronze tools could have facilitated land clearance, cultivation and crop harvesting, technical methods did not differ greatly from the earlier Eneolithic period^20,21^.

The subsistence strategies used by Late Iron Age groups have also been inferred mainly from agriculture but with the addition of several technological innovations that affected human populations over the next centuries: the rotary quern, wheel-turned ceramics and, most importantly, the iron-tipped plough and iron tools that eased plant cultivation, harvesting and processing and thus reduced the overall physical load. Iron age sites under study date to between the 5^th^ century BC and the 3^rd^ century BC (phases La Téne A, and above all La Téne B1–C1)^22,23^.

Further technological improvements in crop processing and livestock breeding followed and the equivalence between such technological innovations and changing lower limb loading can be presupposed^24^. An additional tibial dataset used in this analysis was derived from an Early Medieval agglomeration in Mikulčice (between the 9^th^ century AD and the 10^th^ century AD), Czech Republic, that came from a castle and its surrounding suburban area. The location of the burial ground and grave goods at this site indicate a higher social status for the individuals buried in the castle (i.e. nobles, clergy and military escort), with a considerable proportion of people connected directly with the princely palace. Craftsmen and other individuals involved in the functioning of the stronghold may have been based in the adjacent sub-castle^25^. A further Late Medieval (14–15^th^ century AD) dataset is also used here that originated exclusively from Kutná Hora, a centre of silver/copper mining in the Czech Kingdom, where the mining of silver reached its peak between the 13^th^ century and 14^th^ century. In the middle of the 14^th^ century, the town numbered 18,000 inhabitants and the specific features of a mining town were also reflected in its population development and social structure^26,27^.

A chronologically subsequent sample (our 20^th^ century dataset) is derived from the Pachner anatomical collection and largely comprises people from lower socioeconomic groups who lived under adverse and stressful conditions^28^. Their poor living standards, nutritional hardships and high environmental stress during development are revealed by high fluctuating asymmetry values (in the studies of Kujanová *et al*.^29^ and Bigoni *et al*.^30^), gracility of skeletons^31^ and poor dental health^32^.

In terms of our chronologically youngest group (the 21^st^ century dataset), the socioeconomic context remains unknown and 3D models were derived from anonymized computed tomography (CT) images. This group can nevertheless be described as having experienced the best quality of life regarding nutrition, individual health and degree of stress from different origins. The 21^st^ century tibiae in this case probably represent the lower extreme of the lower limb bone loading spectrum, because life in the 21^st^ century requires a substantially reduced level of mobility and physical load than all previous periods.

This study is based on the documented capability of long bones to adjust their morphology in response to a load to which the bone is subjected. We focus on the curvature of the human tibia, although it is not entirely clear what function this specific skeletal feature actually serves. The study aims to determine whether there is a relationship between tibial curvature and mobility across a diachronic sequence of humans with presumed different mobility patterns within a restricted geographic area. Building on the findings of Macintosh *et al*.^3^, we hypothesized that a decreasing degree of A-P curvature would be predicted with decreasing physical load. We also hypothesized that differences would be observed in medial view and that the same mechanical stimuli that shaped A-P shaft bending may have influenced M-L curvature of the anterior tibial crest (visible in anterior view). We therefore predicted that prehistoric and historic populations in which activity levels were higher would display distinct M-L curvature, as compared with modern samples. Another goal of the study was to test potential effect of tibial size on bone curvature and possible sex-based variation.

## Materials and Methods

### Chronological subsets

A total of 435 3D tibial models from different populations were used for this study to ensure coverage of multiple time periods within similar geographic locations across the Czech territory. The sample was then divided into seven datasets on the basis of time periods, comprising the Eneolithic (the Bell Beaker culture and the Corded Ware culture), the Early Bronze Age (Únětice culture), the Late Iron Age (La Téne culture), an Early Medieval sample, a Late Medieval sample, a 20^th^ century sample and a 21^st^ century sample. Because no radiocarbon dates were available for most sites, our numeric dates were based on approximate relative chronology and the archaeological context of the given site and/or period from across Czech territory. Relevant details on all tibiae from sites and cemeteries included in our analyses are presented in Table 1; for bones excavated from archaeological sites, basic paleodemographic characteristics were taken from data in the archive of the Department of Anthropology of the National Museum, Prague. Sexual classification was verified using Brůžek’s visual method^33^, and age-at-death was estimated by applying a combination of methods from Buikstra and Ubelaker and Schmitt *et al*.^34,35^. All skeletal remains excavated from archaeological sites are housed in the depository of the National Museum, Prague.

**Table 1:** List of samples used in analyses (N, number; M, males; F, females; y., years).

Dataset	Sites (archaeological samples)	N	M	F	20–40 y.	40–60 y.	over 60 y.
Eneolithic	Blšany, Brandýsek, Brozany, Čachovice, Kněževes, Kolín, Konobrže, Kouřim, Krabčice, Lochenice, Most, Obrnice, Praha 5 - Malá Ohrada, Praha 5 - Smíchov, Praha 8 - Kobylisy, Pavlov, Poláky, Postoloprty, Praha - Lysolaje, Prosetice, Stará Kouřim, Široké Třebčice, Tuchoměřice, Velké Přílepy, Vikletice, Vlíněves, Vrbice, Vyškov, Žabovřesky	46	24	22	26	20	0
Early Bronze Age	Blato, Brandýs nad Labem, Březno, Horoměřice, Hoštice, Klecany, Kolín, Moravská Nová Ves, Mořice, Mušov, Olomouc-Slavonín, Praha 5 - Hostivice, Praha 5 - Malá Ohrada, Praha 8 - Čimice, Praha 9 - Čakovice, Pavlov, Praha Liboc, Přibice, Soběsuky, Suchohrdly, Toušeň, Tvarožná, Úholičky, Újezd u Brna, Únětice, Velešovice, Velké Pavlovice, Velké Přílepy, Velké Žernoseky, Vlíněves	79	32	47	38	41	0
Late Iron Age	Hoštice, Jenišův Újezd, Kutná Hora, Kolín, Medlovice, Moravská Nová Ves, Mutěnice, Praha 5 - Jinonice, Pavlov, Praha 6 - Jiviny, Prosmyky, Radovesice	30	20	10	11	19	0
Early Medieval	Mikulčice	103	58	45	44	59	0
Late Medieval	Kutná Hora - Sedlec	57	37	20	40	17	0
20^th^ century		64	33	31	12	30	22
21^st^ century		56	29	27	3	16	37
**Total**		435	233	202	174	202	59

A sample of the 20^th^ century adult tibiae was selected from the Pachner osteological collection, and demographic data were acquired from autopsy records. This skeletal collection, originating from the 1930s and representing poor people from Prague^28^, is curated at The Institute of Anatomy, First Faculty of Medicine, Charles University in Prague. All dry bones (prehistoric, historic and 20^th^ century sample) exhibited full epiphyseal closure, and only left tibiae were digitized for this analysis. We included only normal, non-pathological bone without any indication of injury or advanced degenerative characteristics of senescence in our analysis.

Our most recent group (the 21^st^ century dataset) comprised 3D models of left tibiae from 57 living individuals. Bony surfaces were extracted from clinical anonymized CT scan sequences of adult individuals who had undergone angiography via this approach between 2010 and 2013 (Faculty Hospital Královské Vinohrady, Prague, Czech Republic). CT data were accompanied only by records of age and sex. All groups were represented by relatively equal numbers of males and females (Table 1), and pooled sex samples were used for analysis.

### Collection and processing of scan data

A series of 3D polygonal meshes were obtained via optical scanning of skeletal datasets (dry bones, N = 378) and generated from CT scans (N = 57) of our modern dataset. To digitize dry bones, a smartSCAN 3D-HE scanner (Breuckmann, GmbH, Meersburg, Germany) with a 5 Mpix camera was used; in the system configuration (field of view M-600, 480 mm × 360 mm) we used, the resolution in both the x and y axes was 360 μm. The resulting datasets, imaging the bony surface in four different positions, were then processed and merged using OPTOCAT software (Breuckmann, GmbH, Meersburg, Germany) to make each final data object. After the bony surfaces were scanned and meshes created, they were then exported and saved in .obj format to store 3D graphical object data described by a series of polygons.

Surface models of modern-day tibiae were then created using reconstruction methods using virtual 3D modelling from the Digital Imaging and Communications in Medicine (DICOM) image sequence of CT outputs. CT scanning (Siemens Definition AS+ CT, Siemens, Erlangen, Germany) of the tibiae was conducted using a matrix of 512 × 512 pixels, an X-ray tube adjustment of 120 kV and 51.6 mAs, pixel size of 0.977 mm and slice increment of 0.5 mm (i.e. the standard settings for angiography examinations). Image segmentation of CT data was performed using the software Mimics (Materialise, Leuven, Belgium). All areas of CT scans with a specified range of gray values corresponding to bone tissue were recognized and manually thresholded in the first segmentation step. A series of 3D geometric models were then built using a semi-automatically generated mask, which defined the exterior bone edges precisely, before they were covered with a polygonal mesh surface (.obj format). The suitability of including both scanned and tomographic data has been justified by previous studies^36,37^.

### Semi-landmark data collection

**Figure 1:**
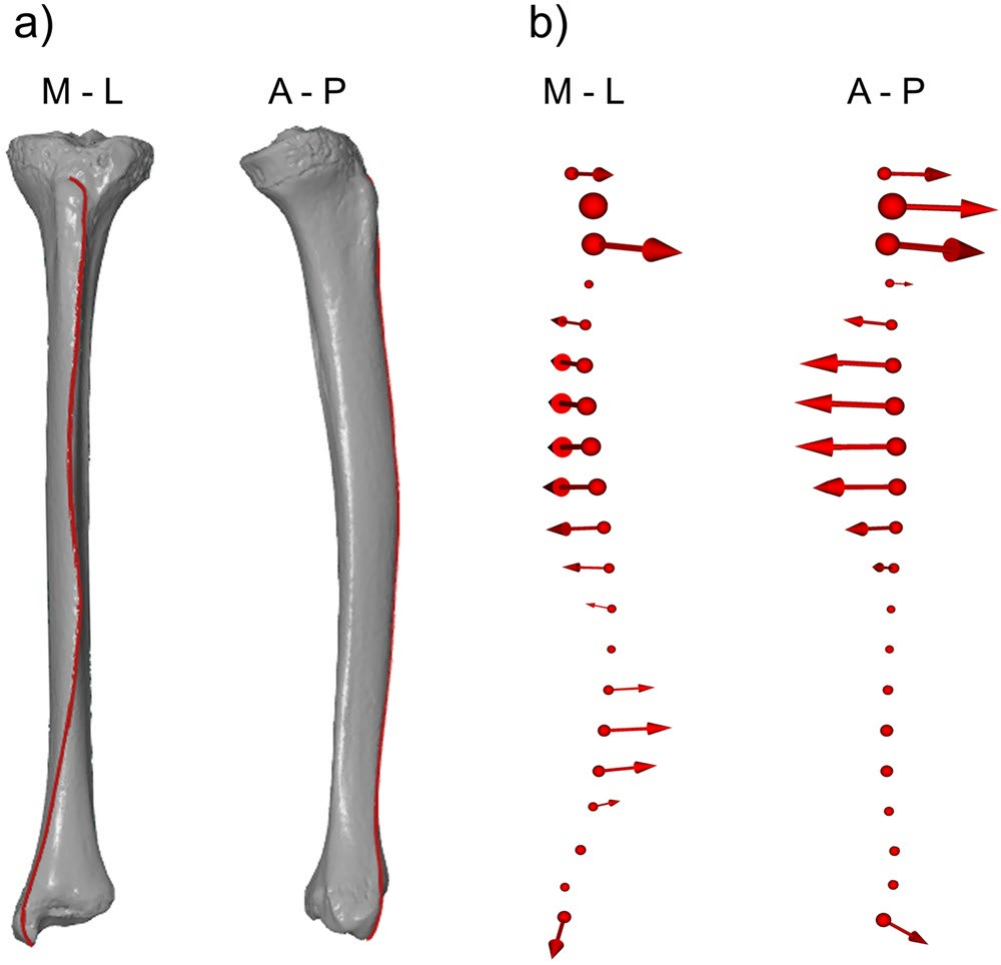
(**a**) Curve placed on a 3D surface mesh of the left tibia in anterior (left) and medial view (right) representing the anterior crest curvature. (**b**) Vector plots contrasting the curvature at the two extremes of the chronological range. Lines indicate the direction of change from Eneolithic to 21^st^ century tibiae.

Semi-landmarks refer to a series of points located along a curve or surface lacking traditional landmarks^38^. Anterior tibial curves were taken on polygonal meshes by a single user (HB) using the software GOM Inspect (Optical Measuring Techniques GOM, Germany). One curve for each tibia was registered, representing the defined anterior crest of the tibia circumscribed by two type III anatomical landmarks^39^ placed on homologous locations and marked before semi-landmark collection. The beginning of each curve was placed at the most proximal point of oval formation of tibial tuberosity, identified with each bone fixed in a vertical position. Curves passed tuberosity and followed the anterior crest with the end located at maximum distal point of the medial malleolus (computed as the most distant point from the medial intercondylar tubercle of the plateau) (Fig. 1a). After a curve was extracted from the software, each was exported as an .asc file listing all of its constituent 3D coordinates (the point sets included from 1,800 points to 2,000 points). A polyline described by the point set was subjected to a series of specific computational steps to define semi-landmark representation of the curve by subdividing it equidistantly by twenty points. This number was chosen experimentally to capture the complexity of shape adequately without introducing too many noise variables.

### Intra-observer error

To test 3D curve extraction repeatability from a surface model, data were recorded repeatedly from 15 bone models. In this process, each specimen was landmarked three times, including a time interval of at least 3 days between each individual measurement, and error was evaluated as the standard deviation of N measurements of an individual landmark^40^. Overall intra-observer measurement error was an average of all the measurement errors of the full semi-landmark configurations derived from landmark measurements. The fraction of overall measurement error and data standard deviation are the root of the coefficient of reliability, a single value on the scale from 0 to 1, indicating amount of error in data with an acceptable 0.95 threshold.

### Morphological comparisons

A range of methods for quantifying bone curvature have been proposed^41–51^. In this study, a set of curves in semi-landmark format was processed using the geomorph R package, as proposed by Adams *et al*.^52^. Using this approach, we altered the positions of semi-landmarks into new positions according to sliding extension to standard Generalized Procrustes Analysis^39^, minimizing Procrustes distances across the sample. Using this method, shape information was extracted from semi-landmarks by stripping it from difference in position, orientation, scale and subtle differences in position along the curve. Procrustes distance minimization was used here rather than bending energy minimization for sliding semi-landmarks, with the exception of the first and the last entries on a curve, in directions given by an adjacent pair of semi-landmarks, because this procedure gives more plausible visual results without extreme changes in semi-landmark position.

Overall shape variability was then deconstructed into orthogonal principal components and studied independently by using Principal Component Analysis (PCA)^39,53^. This process enabled us to reduce the dimensionality of the dataset and explore the placement of individuals and groups within the shape space. Statistically significant differences between chronologically distinct groups of shapes were determined using permutation variants of two-sample Hotelling’s T^2^ test^54^ on the shape variables; the threshold of significance was taken as α = 0.05 in this study, and the number of statistically significant shape variables was determined using a broken-stick criterion^55^.

In cases where significant group differences were detected, we accomplished spatial visualization of mean shapes by using vector plots. These plots display the directional change required to relocate the semi-landmark locations of one (reference) configuration onto the corresponding semi-landmark locations of a second (target) configuration. Information about group shape differences was then expressed as the magnitude of the difference between group means and visualized as projections with spheres instead of 20 semi-landmarks, and their diameters reflected magnitudes. The same procedure was performed for directions of local shape differences and visualized semi-landmark shifts from one group mean to the other were denoted with a 3D arrow extending from the sphere (Fig. 1b).

To detect potential subgroups differences attributable to sexual dimorphism, all morphological comparisons were also conducted in subsamples created based on sex. To control for the potential effect of size on tibial anterior curvature throughout the period, allometry was evaluated by looking for linear relationships between size (curve length) and shape variables (PC score). Statistical significance of the model was assessed by Analysis of Variance (ANOVA).

Maximum tibial length was measured directly from meshes by using the book distance tool in the software Morphome3cs (v. 2.0, Faculty of Mathematics and Physics, Charles University, Prague)^56^. We used the software R (version 3.4.4) (R development Core Team)^57^ and custom-made tools written in Python (version 3.6.5) using the VTK library (version 8.0.0) to enable statistical analyses and the visualization of results.

## Results

We conducted an initial error study to verify whether curve placement consistently captured the same shape information. The results were confirmed by means of the reliability coefficient (σ_xx’_ = 0.98)^40^.

**Figure 2:**
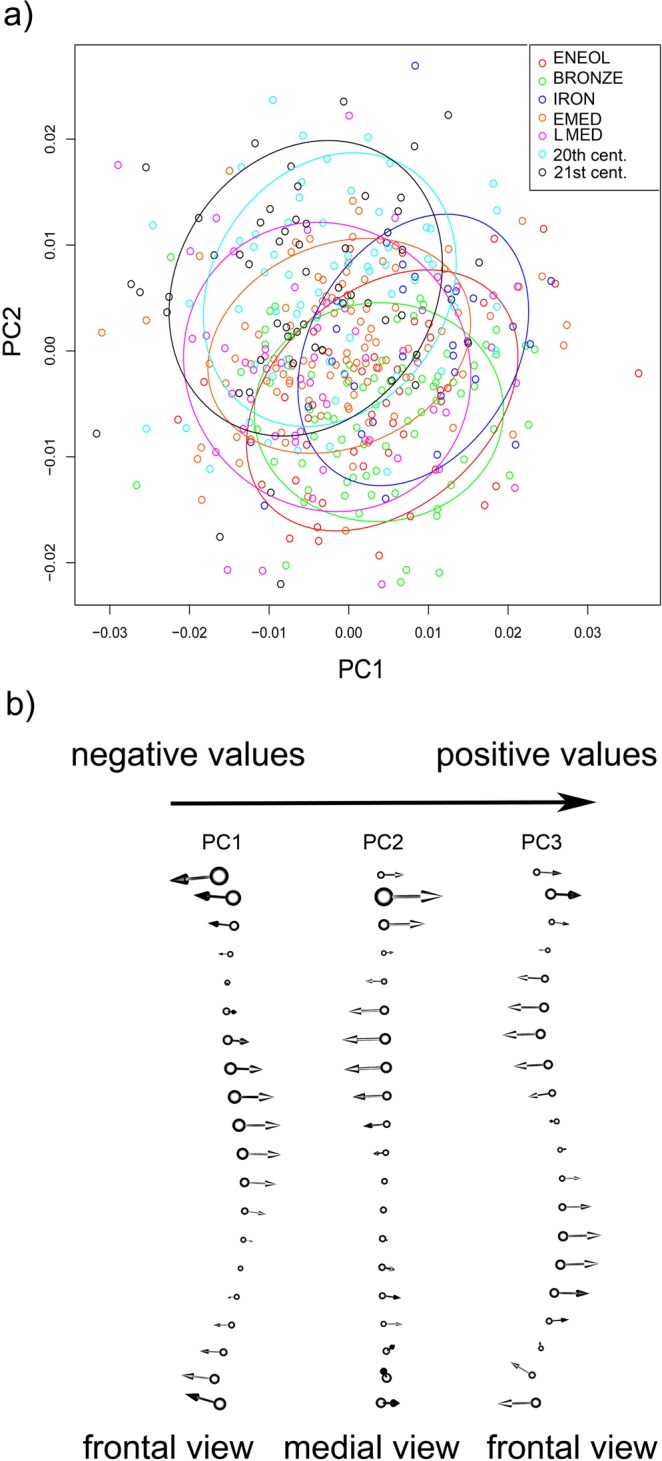
(**a**) Scatter plot showing the positions of individuals plotted on PCs 1 and 2. Diachronic groups are coded by chronological age and delineated with 70% confidence ellipses. (**b**) The effect of PC1-3 on tibial anterior crest curvature. Abbreviations: ENEOL, Eneolithic; BRONZE, Early Bronze Age; IRON, Late Iron Age; EMED, Early Middle Ages; LMED, Late Middle Ages; 20^th^ cent., 20^th^ century group; 21^st^ cent., 21^st^ century group.

Our PCA results for shape variables revealed a substantial overlap between chronologically distinct groups (Fig. 2). PC1 (accounting for 33.6% of the shape variation) appears to primarily describe the degree of M-L curvature visible in anterior view. Low PC1 loaders match to modern groups with more sigmoidal anterior crest curvature. The beginning of each curve in the highest PC1 loaders (prehistorical groups) is directed more medially, and the middle third of the curve aims more laterally, resulting in a less S-shaped contour. PC2 accounts for 25.7% of sample shape variation and describes a change in A-P curvature that can be seen in medial view. The lowest scores in this case belong to members of prehistoric groups with more pronounced A-P curvature, and individuals with higher PC2 scores tend to have tibiae with straighter shafts than their counterparts from chronologically older datasets. The effect of PC3 (not displayed in our scatter plot, and accounting for 7.4% of shape variation) is visible in anterior view and involves the whole curve with more accentuated laterally concave and convex course in modern groups with highest scores.

Although diachronic groups cannot be clearly separated in our scatter plot (Fig. 2), our second set of analyses nevertheless identified numerous statistically significant chronology-based differences between datasets using shape variables. These are summarized in Table 2 via p-values of permutation tests, which reveal a statistically significant separation between all groups, with the exception of the temporally adjacent Eneolithic versus Bronze Age, Early versus Late Middle Ages, and 20^th^ century versus 21^st^ century comparisons (Table 2).

**Table 2:** Summary of p-values indicating the statistical significance of diachronic group differences in 3D anterior tibial crest curvature, assessed using Hotelling’s T^2^ test with 10,000 permutations (significance level of p < 0.05, displayed in bold) (abbreviations as in Fig. 2).

	ENEOL	BRONZE	IRON	EMED	LMED	20^th^ CENT.
BRONZE	0.486					
IRON	**0**.**019**	**<0**.**001**				
EMED	**<0**.**001**	**<0**.**001**	**<0**.**001**			
LMED	**0**.**017**	**<0**.**001**	**<0**.**001**	0.394		
20^th^ CENT.	**<0**.**001**	**<0**.**001**	**<0**.**001**	**<0**.**001**	**<0**.**001**	
21^st^ CENT.	**<0**.**001**	**<0**.**001**	**<0**.**001**	**<0**.**001**	**<0**.**001**	0.182

**Figure 3:**
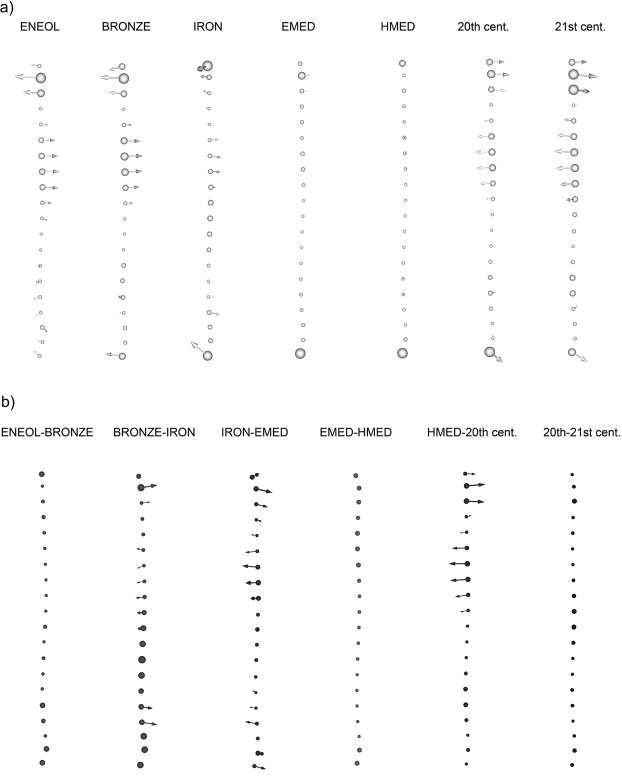
(**a**) Shape differences in particular samples with respect to chronological age. Medial view of left anterior tibial curvature, with the arrows showing the A-P shape change from the mean curve of pooled sample toward the mean curve of the chronologically specified dataset. (**b**) Vector plot showing the shape differences in the left anterior tibial A-P curvature between chronologically adjacent groups. Chronologically older diachronic groups are represented by circles and chronologically younger samples by arrow points (abbreviations as in Fig. 2).

Averaging semi-landmark coordinates enabled the development of a mean curve shape for each dataset that was initially compared to mean shape from the whole sample. The 3D views, as seen from the side (medial) view, display and localize the site of the shape changes and the manner in which the anterior tibial crest straightened in an A-P direction over time. Our Eneolithic, Bronze Age and Iron Age samples all contain tibiae that are more anteriorly convex than the mean pooled sample curve, and both Medieval datasets coincide with the mean of the pooled sample. Both modern groups display an anterior shift in the beginning of each curve with a simultaneous posterior shift of the second proximal curve quarter, resulting in an anterior crest that is straighter than the mean. In general, prehistoric datasets tend to be characterized by more pronounced curvature in the A-P plane than that of their Medieval and modern counterparts, mainly because of changes in the upper half of the shaft (Fig. 3a).

A visual comparison of vector plots directly comparing the mean shapes of temporally adjacent groups reinforces the similarity in curvature between Eneolithic and Bronze Age samples, as well as our two Medieval and two modern datasets, in which statistically significant differences were not found (Table 2, Fig. 3b). Bronze Age versus Iron Age comparison revealed only a slight anterior shift in the curve beginning of the chronologically younger dataset, and the subsequent time period examined (Iron Age versus Early Medieval) is characterized by a more marked anterior shift of the beginning and posterior shift of the second proximal curve quarter with time. A further statistically detectable change was found in the case of our Late Medieval versus 20^th^ century comparison, in which analogous change can be seen validating the continuous trend of A-P straightening in initially more curved shafts over time.

**Figure 4:**
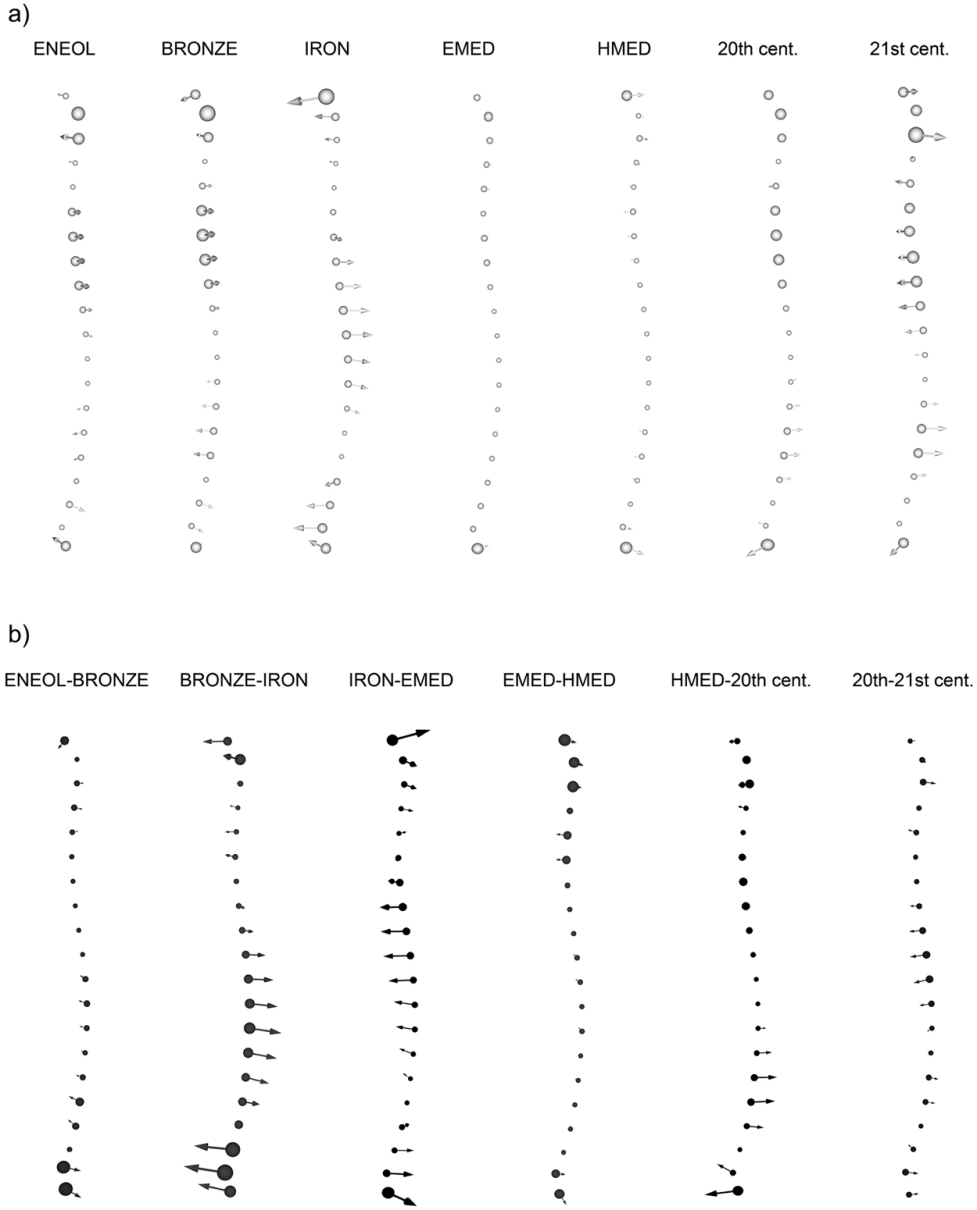
(**a**) Shape differences in particular samples with respect to chronological age. Anterior view of the left anterior tibial curvature, with arrows showing the M-L shape change from the mean curve of pooled sample toward the mean curve of the chronologically specified dataset. (**b**) Vector plot showing the shape differences in left anterior tibial M-L curvature between chronologically adjacent groups. Chronologically older diachronic groups are represented by circles, and chronologically younger samples are represented by arrow points (abbreviations as in Fig. 2).

The additional analyses which we performed related to variation in the M-L curvature. Visualizations of each group mean shape versus mean shape of the pooled sample displayed in anterior view are shown in Fig. 4(a); in this case, larger spheres distinguishing the most divergent regions indicate that the M-L curvature varied visibly through time. The Eneolithic mean longitudinal curve differs from the mean shape via less pronounced lateral concavity in the upper half of the shaft. The upper half of the curve is less laterally concave in our Bronze Age sample and is accompanied by a slight medial shift in the start of the curve and a slight weakening of lateral convexity over the lower half of the tibial length. The major difference between Iron Age sample and mean shape relates to the finding that the central portion of the curve shifts somewhat laterally and that the start and end of the curve shift medially. Early and Late Medieval curves coincide with the mean curve shape of the pooled sample, apart from lateral shifts at the ends in each case, whereas our 20^th^ century dataset is characterized by faint accentuation of lateral convexity in the lower half of the curve. The anterior crest is more laterally concave in its upper half and more laterally convex in its lower half than in the mean curve within our 21^st^ century group (Fig. 4a).

Similarities and differences between temporally adjacent datasets are shown in Fig. 4(b). It is noteworthy that from the Eneolithic to the Bronze Age, our M-L curve does not change markedly, in accordance with statistical significance tests, and the limited effect of chronology is also apparent in the absence of temporal differences between both Medieval and modern datasets (Fig. 4, Table 2). In the first place, between-group transitions in morphology are clear in the relatively more laterally convex lower half of the Iron Age curve compared with our Bronze Age group, whereas the Early Medieval curve differs from the Iron Age curve in the presence of a medial shift visible on the central portion of the shaft that partly accentuates upper lateral concavity and simultaneously moderates lower lateral convexity. During the Middle Ages, the curve remains identical, whereas the transition from Late Middle ages to the 20^th^ century requires just a change in lower half lateral convexity, which is more marked in the modern group. In contrast to our observations of A-P shaft curvature, these results suggest a trend of increased M-L curvature in modern groups compared with prehistoric and Medieval datasets. We therefore tested the chronologically oldest (Eneolithic) and youngest (21^st^ century) groups and generated a plot that corroborates this temporal trend by using both extreme shapes (Fig. 1b).

Statistically significant differences between males and females are observed only in the Early Medieval (p < 0.001) and in the 20^th^ century sample (p = 0.018). Comparison of temporally distinct male and female groups yields general information regarding the significance of chronology-based differences between sex groups (Supplementary Information Table S1). Note that results often become non-significant when subsamples are created based on sex, especially in female groups. Even so, considered and visualised separately, the vector plots showing the shape differences in the A-P and M-L planes in particular sex groups (Supplementary Figs S1–4) are almost identical to the visualisations made from sex-pooled data (Figs 3 and 4). Only 21^st^ century female tibial curve exhibits a more pronounced trend of A-P shaft straightening and M-L curve distortion than the mean curves derived from the 21^st^ century male or sex-pooled (but statistically significant difference between sexes was not found in this particular sample, p = 0.091) (Supplementary Figs S3a and S4a).

A comparison of vector plots contrasting the mean shapes of temporally adjacent sex groups also reveals a more pronounced trend of A–P straightening between Iron Age and Early Medieval male groups (Supplementary Fig. S1b). The Iron Age vs. Early Medieval comparison also unmasks a more pronounced medial shift of the central portion of the M–L curve in Early Medieval females (Supplementary Fig. S4b) than can be seen in male vector plots (Supplementary Fig. S2b). Other sex-based differences are identified in Late Medieval vs. 20^th^ century comparisons where changes in morphology are more obvious in females. When the differences between Late Medieval and 20^th^ century sex-groups are depicted graphically, chronologically younger females exhibit a more pronounced trend of A–P shaft straightening (Supplementary Figs S1b and S3b). In addition, 20^th^ century males are characterised by a more pronounced lateral shift of the lower half of the M–L curve in comparison to females of the same chronological age (Supplementary Figs S2b and S4b).

Nevertheless, sex groups had significantly distinct morphologies in only our Early Medieval and 20^th^ century samples and, for this reason, we generated plots contrasting mean male and mean female curves acquired from these temporal datasets. Early Medieval females differed from Early Medieval males in the presence of the posterior shift of the curve beginning (visible in the A-P plane) and in lateral shift of the same semi-landmarks apparent in the M-L plane (Supplementary Fig. S5a). Female curve was also slightly more anteriorly convex (see A-P vector plot in Supplementary Fig. S5a) and its central portion shifted more medially in the M-L vector plot relative to males of the same chronological age. 20^th^ century females differed from 20^th^ century males in the presence of a minor anterior shift of the curve start visible in the A-P vector plot and in the less sigmoidal anterior tibial curve apparent in the M-L plane (Supplementary Fig. S5b).

**Figure 5:**
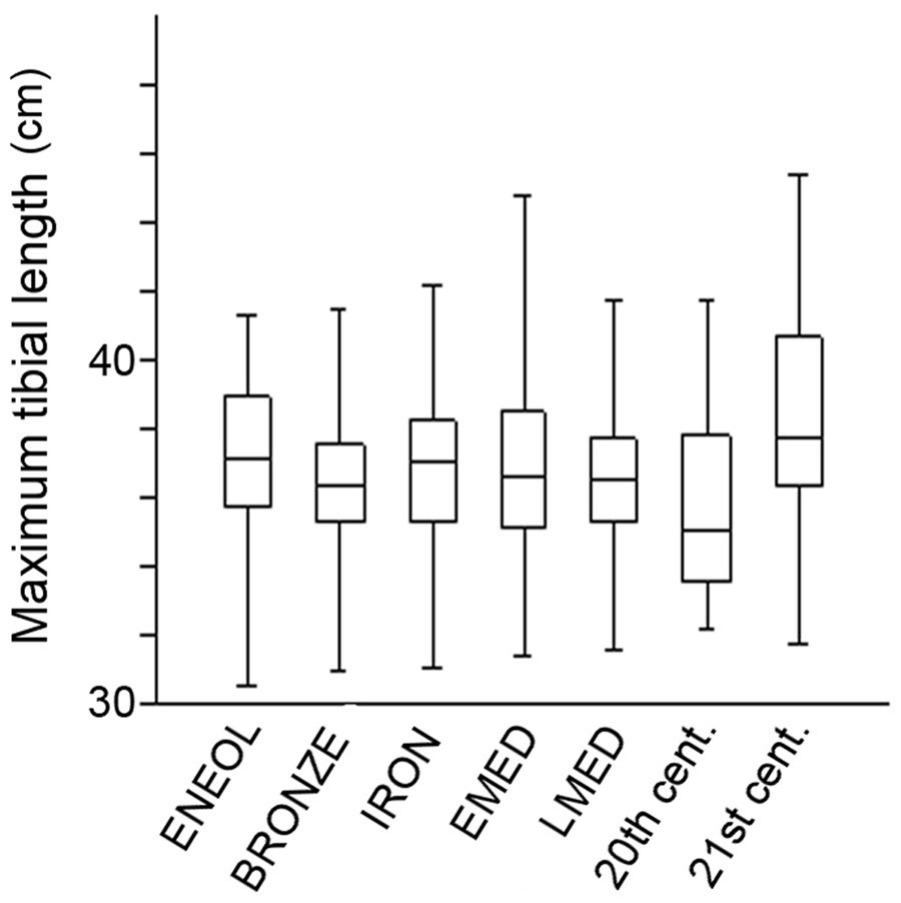
Variation in maximal tibial length between chronologically diverse populations. For each group, the 25–75 percent quartiles are drawn using a box, the median is shown with a horizontal line, and the minimum and maximum values are shown with whiskers (abbreviations as in Fig. 2).

The variation in maximal tibial length between chronologically diverse datasets is shown in Fig. 5. This variable did not change significantly over the relatively long time-span between the Eneolithic and Late Middle Ages, but then decreased in the subsequent period from the Late Middle Age to the 20^th^ century, and then increased at the end of the time range examined between the 20^th^ century and the 21^st^ century (Fig. 5; Table 3). The mean tibial length across our study populations showed a faint, but positive, temporal trend, with one negative deviation in the 20^th^ century dataset heavily weighted toward members of low socioeconomic groups. The absence of allometry (interaction between size and shape) was verified for each sex-group using ANOVA performed on linear models of the curve length as an indicator of size and predictor, and on PC scores as indicators of shape features and response (Supplementary Table S2).

**Table 3:** Summary of p-values indicating the statistical significance of diachronic group differences in maximal tibial length.

	ENEOL	BRONZE	IRON	EMED	LMED	20^th^ CENT.
BRONZE	0.26					
IRON	0.65	0.66				
EMED	0.68	0.48	0.90			
LMED	0.21	0.74	0.53	0.36		
20^th^ CENT.	**0**.**01**	**0**.**05**	0.08	**0**.**02**	0.14	
21^st^ CENT.	**0**.**02**	**<0**.**001**	**0**.**01**	**0**.**001**	**<0**.**001**	**<0**.**001**

To test for differences between maximal lengths, permutation tests with 10,000 replicates were performed (significance level of p < 0.05, displayed in bold) (abbreviations as in Fig. 2).

## Discussion

The results of this study suggest that anterior crest curvature is a component of tibial design which shows a consistent temporal trend accompanying presumed decreasing mechanical forces exerted on the human lower limb. As introduced, previous attempts to study human lower limb bone curvature have focused predominantly on the femur; Shackelford and Trinkaus^1^ and De Groote^2^ documented temporal declines in the degree of femoral A-P curvature and noted that maximum levels were found in samples with assumed highest mobility levels. In terms of the tibia, Macintosh *et al*.^3^ documented a steady decline in tibial A-P curvature through time from the Neolithic to Middle Ages in preindustrial Central European groups. Both the tibial A-P curvature and the tibial shaft cross-sections mirrored the presumed decrease in mechanical load on the tibia in populations experiencing prolonged cultural change.

The semi-landmark methods applied in this study provide evidence regarding temporal changes in greater detail together with changes identifiable in the M-L plane. The results of our present study expand the chronological and lifestyle range of known samples by adding two modern industrialized populations; our results are consistent with those from previous investigations^3^ and reveal significant changes between almost all diachronic datasets. This research investigated temporal changes in tibial curvature including the periods of recent urbanization and industrial development by incorporating a larger number of samples.

Ancient population activity patterns can be only roughly estimated, and all the archaeological populations under study shared a similar base subsistence strategy with primary reliance on domesticated crops and livestock. When assessing an assemblage as a whole, archaeologists can draw conclusions about the acquisition of food and other resources from settlement finds and settlement features (such as storage pits), grave goods, house constructions, type of ceramics and botanical and faunal macro- and micro-remains^20–23^.

The better-identifiable changes through the time period explored in this study are only the major changes in technology–both ancient and recent–that could have markedly decreased the level of loads imposed on the lower limbs^24^.

No significant 3D curve shape differences were found in three particular cases, as our Eneolithic group was indistinguishable from the Bronze Age group, the Early Medieval sample was indistinguishable from the Late Medieval sample, and the 20^th^ century group was not distinct from the 21^st^ century group. In view of the overall similarity between the aforementioned groups, these chronologically adjacent populations appear to have had similar lifestyles, with similar biomechanical loads placed on the lower limbs.

The Eneolithic individuals exhibited the same A-P and M-L curvature as Early Bronze Age agriculturalists, thus corroborating the results from the earlier biomechanical studies of Sládek *et al*.^18,19^. Lower archaeological visibility of Eneolithic groups has commonly been interpreted as a characteristic of communities with higher mobility incorporated into their subsistence strategies^20^. In spite of the fact that daily activities would have become less evident in Late Eneolithic Central Europe, biomechanical analyses of the tibial and femoral shafts did not support an extremely high degree of overall mobility during this span in comparison with later populations^18,19^. In agreement with direct biomechanical approaches, our semi-landmark techniques found no substantial shape differences in tibial anterior curvature signalling change in approximate loading profile.

The lifestyles of our Late Iron Age group indicated very similar levels of A-P tibial curvature as preceding samples with slight straightening of upper half of the shaft; over this particular time-span, changes in habitual behavior were expected, owing to the socioeconomic transformations and more heterogeneous societies that characterized this period. The Late Iron Age sites under study represent mostly agricultural communities, and a subset of the population may have been engaged in specialized production, craft industries, iron metallurgy, smithery, or wood processing^23^. In any case, a major change in technology, intensive exchange of technological knowledge and further differentiation of work had decreased the level of loads imposed on the lower limbs to such an extent that they impacted both the tibial cross-sectional geometry^24^ and the anterior curvature.

More distinctive changes in A-P curvature occurred between the Late Iron and Early Medieval periods, when Early Medieval tibiae became straighter in the A-P direction than chronologically older ones. Surprisingly, however, despite increasing urbanization, our Early Medieval sample is not significantly different from the Late Medieval group; on the whole, Early Medieval individuals (Mikulčice site) lived under favourable conditions, a conclusion validated by studies of skull shape asymmetry^30,58^. Our sample might therefore be composed of nobility, members of a military entourage, servants, craftsmen and farmers. Thus, taking into consideration the archaeological facts relating to locality, this particular sample might be associated with relatively high physical loads and mobility levels, as individuals performed activities such as walking for long distances, farming, horseback riding, carrying heavy loads (wood) and frequent weapon use^59^. In the Late Medieval dataset (derived from the Kutná Hora site), the most numerous population group was workers employed in mines, foundries, ore washers, coal depots and transport. In this case, not only male but also female workers were employed in washing and sorting ore. In addition to the other professions necessary for the functioning of mines, foundries, metallurgy and mints, contractors, artisans, merchants and persons providing a constant supply of food, wood and brown coal also lived within this region^26^. Despite a substantial increase in population density and the correlated assumption that living conditions would have worsened due to the intensity of mining activities, it seems that Early medieval and Late medieval town lifestyles shared similar lower limb loading regimes. The only common feature of our medieval populations, which would impact on overall mobility levels, is that they were mostly or entirely dependent on supplies from the suburbs or agricultural hinterland^26,59^.

Subsequent diachronic comparisons (Late Middle Ages versus 20^th^ century sample) revealed further tibial shaft A-P straightening caused by an anterior shift of the beginning and a posterior shift of the second proximal curve quarter with time. The character and level of habitual daily activities of the 20^th^ century dataset remain unclear, however; in general, anatomical collections may not be representative of the population, because they tend to be biased by economic status^60^. Moreover, the Pachner collection is heavily weighted towards low socioeconomic groups. These individuals represent poor people from Prague (Czech Republic) in the 1930s^28^ with an assumed deficiency in their diet and high levels of environmental stress as reflected by various skeletal indicators: skeletal asymmetry^29,30^, gracility^31^ and poor dental health^32^. Poor living standards, in particular during the childhood and adolescence of these individuals, could have caused considerable disruption to tibial growth. Nevertheless, 20^th^ century tibiae tend to exhibit curvature identical to that of their 21^st^ century counterparts experiencing substantial improvements in their living conditions. We therefore suppose that one crucial factor involved in straightening of tibial A-P curve was industrialization, and both modern samples tended to be the least mobile. In summary, earlier (prehistoric and medieval) populations whose members devoted more effort to scouting or acquiring food resources and raw materials tend to exhibit more A-P curved tibiae than those observed in modern times. And vice versa, modern populations with presumed lower activity levels tend to exhibit lower degrees of A-P curvature than chronologically older groups. A general assumption that can be made from our medieval and modern samples is that the overall lower limb loading regime appears to be a more significant determinant of temporal variation than living conditions.

Because the anterior border of the human tibial shaft also follows a laterally concave/convex course down the front of the shaft, it was expected that temporal trends described for A-P curvature^3^ would also be accompanied by corresponding changes in M-L direction. On the basis of data collected from five archaeological skeletal series and two modern datasets, we confirmed that anterior tibial border changes with time also occur in the anterior view. As is the case with A-P curvature, correspondences and similarities among 3D digitisations of the anterior tibial crest were observed in chronologically adjacent pairs of datasets, especially in populations undergoing similar economic and behavioral transitions. This phenomenon was observed in Eneolithic versus Bronze Age comparisons as well as in Early versus Late Middle Ages and in 20^th^ century versus 21^st^ century group comparisons in which no significant 3D curve shape differences were found. Statistically significant between-group differences showed less consistent temporal trend compared to the steady curve straightening apparent in the A-P plane.

Contrasting M-L curves at the two extremes of the chronological range revealed that the initially less curved crest accentuated both its lateral concavity in the upper half and lateral convexity in the lower half of the limb between the Eneolithic and the present day (Fig. 1b). In groups of active walkers or runners the tibia shows greater bending stresses in the A–P than the M–L plane due to the traction of the calf and thigh muscles (soleus, gastrocnemius, and quadriceps femoris) on the tibial shaft^61^. It appears that the M–L curvature becomes accentuated only under certain conditions, i. e. when these local stresses and A–P strains weaken. Thus, modern diachronic groups with lower A-P tibial curvature tend to display more pronounced M-L curvature, and these morphological features can be assigned to skeletal changes associated with sedentism, urbanisation, industrialisation, decreased mobility and diminished physical work load. An association between A-P straightening and M-L curve accentuation was also recorded in our earlier surface-based study^62^ focusing on temporal variation of sexual dimorphism. To better visualise sex-specific morphological features in our previous research, we created extreme female and extreme male tibial shapes, i.e. artificially generated surface models (30 times sigma from the mean shape) in which the 21^st^ century female tibiae were generally straighter when viewed from the side and more curved in the anterior view than those of 21^st^ century males^62^.

This brings up the question as to whether males and females develop tibial anterior curvature in the same way or to the same extent under the same level of loading. There is strong evidence from our previous studies of significant sex differences in the shape of tibial articular ends^63,64^ along with sex-specific temporal trends in whole-bone geometry of tibiae derived from Czech populations dated from the Early Middle Ages to the present day^62^. This temporal variation could be connected to changes in living conditions and presumed decrease in lower limb loading/labour division in the last 12 centuries having the greatest effect. In the current study, using semi-landmarks located along the anterior tibial crest, we observed statistically significant sex-based shape differences surprisingly only in the Early Medieval and in the 20^th^ century samples. When exploring chronology-related differences between male and female sub-groups, statistically significant differences decreased considerably in number in the female groups (Supplementary Table S1). Despite this decrease (probably caused by smaller sample sizes), there was no striking change in vector plots showing the temporal changes either in the A–P or in the M–L plane (Supplementary Figs S1–4). Both in males and in females, we observed a similar pattern, where the tibial A–P curve straightened and the M–L curve flexed over time. Among females, the temporal A–P change was less pronounced from the Iron Age to the Early Middle Ages than among males but was more pronounced from the Late Middle Ages to the 20^th^ century (Supplementary Figs S1 and S3). If we assume that A–P curve straightening reflects diminishing lower limb load, a weaker change might indicate a smaller decrease in loading when compared to males in the first time-span cited. These results are partially consistent with the only bioarchaeological study of tibial A–P curvature undertaken to date, where Macintosh *et al*.^3^ reported less strong but steady temporal straightening among females when compared to males from the Neolithic period to the Early Middle Ages. Our findings pointed to the opposite situation in the time span from the Late Middle Ages to the 20^th^ century, with a possible greater impact of technological changes of this time period on women than on men.

A closer look at shape differences in chronological sex-groups with significantly distinct morphologies revealed that sex-based differences were subtle and occurred rather nearer the articular ends than in the course of the anterior tibial curve (Supplementary Fig. S5). Taken together, it may be said that differences due to sexual dimorphism were not strong enough to totally mask the differences attributable to chronology. Semi-landmark analyses of human tibial anterior curvature in this study were able to reflect only the presumed decrease in overall physical effort involving the lower limbs, but not the universal presence of gender-based division of labour^61,65^.

Human lower limb long bone morphology may be influenced not only by the activity performed, but also the topography upon which the activity is carried out and climate^2,64,66,67^. Our re-examination of additional features previously demonstrated to significantly affect long bone shape shows that the studied samples come from areas with similar topography, falling into either flat or moderately hilly landscape categories^16,21,23^. The similarity of physical geography within the same geographical region is accompanied by negligible fluctuations in climatic conditions during the time span covered by our samples^68^. We therefore conclude that only a minimal (if any) part of the variation seen in inter-population differences in tibial anterior crest curvature is related to climatic or topographic variables.

Despite the fact that the samples we studied were geographically identical, we cannot rule out population movements and related gene flow over time. The geographical setting of the samples in the Central European region was definitely associated with repeated migratory events and multiple episodes of population replacement. For this reason, a potential genetic component to the observed temporal trend cannot be disregarded. Genetic factors are assumed to alter the degree to which bones reflect loading history, but experimental data underscore the strong influence of genetics on bone structure and the complexity by which mechanical stimuli may cause such alterations^69^. Likewise, the results of von Cramon-Taubadel *et al*.^70^ suggest that relative human limb dimensions are not tracking the same demographic population history as the human skull and point to the strong influence of non-genetic factors in determining limb bone morphology. Based on these results and on the documented relationship between bone curvature and activity level and mobility^1–3^, we can assume that most of the observed temporal variation in tibial curvature is derived from non-genetic influences. We must also bear in mind the fact that the tibia is part of a complex with the fibula and that studies focusing on the tibia alone may provide an incomplete picture of leg functional anatomy^67,71^. Taking this into consideration, future studies should also address the fibular shape and robusticity and the anatomical position of the fibula relative to the tibia.

A non-genetic factor that might also influence tibial curvature is individual aging. An important limitation in the research described here is that the results may have been affected by an imbalance in age cohorts, but it remains unclear to what extent and in what manner. The prehistorical and medieval groups comprised young (20–40 years) and middle-aged (40–60 years) adults, whereas the 20^th^ century group included a substantial proportion of adults aged over 60 years at death. In addition, in the 21^st^ century group adults over 60 years of age at the time of CT examination accounted for more than half of the sample (Table 1). Although research utilizing samples encompassing a more balanced age distribution would be beneficial, it would have been extremely difficult to realize this in practice. The small proportion of young and middle-aged individuals sampled in the 21^st^ century group was caused by the unavailability of CT scans for these age cohorts and it would have taken many years to collect satisfactory numbers. Due to the novelty of the method, there are no studies which are directly comparable to our findings, but there are enough data available concerning age-related changes of bone at both microstructural and macrostructural levels^65,72–75^. In light of previous findings corroborating age-related changes of the shaft cross-sections, it can be presumed that the bone shaft can moderately change in shape as individuals age^65,72^. Consequently, the possible effect of age should be taken into account when evaluating differences between pre-industrial and industrial samples which could have been accentuated/masked by aging.

Given the time frame of the seven chronological samples under study, the question arises as to whether some part of the variation in tibial anterior crest shape might be accounted for secular increase in body height (reflecting in maximum tibial length). However, there were no significant differences in mean maximum tibial lengths over a relatively long time-span from the Eneolithic to the Late Middle ages (Table 3, Fig. 5). Subsequently, tibial length decreased, and the 20^th^ century tibiae (heavily weighted toward members of low socioeconomic groups) were significantly shorter than those derived from Eneolithic, Bronze Age and Early Medieval datasets. At the end of the time range examined (from the 20^th^ to the 21^st^ century) tibial length increased, and the 21^st^ century tibiae were significantly longer than those from preceding centuries/millennia. Because the 20^th^ century tibiae are, on average, the shortest, and the 21^st^ century tibiae are, on average, the longest, and the anterior 3D curves of samples from these two adjacent groups do not differ significantly (Table 2), variation in tibial size does not explain temporal changes in curvature shape in our sample. The absence of allometry (interaction between size and shape) was verified for each sex-group using ANOVA performed on curve length regressed against shape variables, which showed no significant interaction between shape and size in sex-groups of diachronic samples (Supplementary Information Table S2). These findings corroborate the results of Mactintosh *et al*.^3^, who found no significant correlation between A-P centroid displacement and tibial length, suggesting a minimal influence of body size on curvature in the tibial shaft. Analogously, Shackelford and Trinkaus^1^ in their traditional morphometric study concluded that variation in femoral curvature appears not to be strongly influenced by body size. De Groote^41^ drew the same conclusion from semi-landmark analyses of anterior femoral curvature, demonstrating that neither the magnitude of curvature nor the position of the apex of the curve is allometrically related to size.

The results of this study encourage the continued use of 3D display technologies and geometric morphometrics to precisely capture the contours and other variations notable in bony structures. Comparison of prehistoric skeletal series dating to the Late Eneolithic, Early Bronze and Iron Age, with two Medieval and two modern samples, provided evidence that curvature of the anterior crest in the human tibia has continued to evolve over the past five millennia. We demonstrated a continuous decline in A-P curvature beginning in the Eneolithic/Bronze Age period and advancing to the present day. Interestingly, this adaptive change in bone geometry was associated with a relative sigmoidal curve accentuation in the M-L plane visible in comparison of the two extreme mean shapes of the chronological range. Both of these morphological features can be assigned to skeletal changes associated with sedentism, urbanization, industrialization, decreased mobility and diminished lower limb loading regime over the time-span explored.

## Supplementary information

Three-dimensional geometry of human tibial anterior curvature in chronologically distinct population samples of Central Europeans (2900 BC – 21st century AD)

## Data Availability

The datasets analysed during this study are available from the corresponding author on request.

## References

[1] Shackelford LL, Trinkaus E (2002). Late Pleistocene human femoral diaphyseal curvature. Am. J. Phys. Anthropol..

[2] De Groote, I. A comprehensive analysis of long bone curvature in Neanderthals and modern humans using 3D morphometrics (Ph.D. Thesis). (University College, London, 2008).

[3] Macintosh AA, Davies TG, Pinhasi R, Stock JT (2015). Declining tibial curvature parallels ~6150 years of decreasing mobility in Central European agriculturalists. Am. J. Phys. Anthropol..

[4] Yamanaka A, Gunji H, Ishida H (2005). Curvature, length, and cross-sectional geometry of the femur and humerus in anthropoid primates. Am. J. Phys. Anthropol..

[5] Lanyon LE (1980). The influence of function on the development of bone curvature. An experimental study on the rat tibia. J. Zool.

[6] Bertram J, Biewener A (1988). Bone curvature: Sacrificing strength for load predictability?. J. Theor. Biol..

[7] Main RP, Biewener AA (2004). Ontogenetic patterns of limb loading, *in vivo* bone strains and growth in the goat radius. J. Exp. Biol..

[8] Hall, S. J. *Basic Biomechanics*. (McGraw-Hill, 2004).

[9] Lanyon LE, Bourn S (1979). The influence of mechanical function on the development and remodeling of the tibia: An experimental study in sheep. J. Bone Joint Surg. Am.

[10] Milne N (2016). Curved bones: an adaptation to habitual loading. J. Theor. Biol..

[11] Frost, H. M. *Laws of Bone Structure*. (Charles C Thomas, 1964).

[12] Pauwels, F. *Biomechanics of the Locomotor Apparatus*. (Berlin: Springer-Verlag, 1980).

[13] Frelat MA, Katina S, Weber GW, Bookstein FL (2012). Technical note: A novel geometric morphometric approach to the study of long bone shape variation. Am. J. Phys. Anthropol..

[14] Swartz SM (1990). Curvature of the forelimb bones of anthropoid primates: Overall allometric patterns and specializations in suspensory species. Am. J. Phys. Anthropol..

[15] Carlson, K. J. & Marchi, D. Introduction: towards refining the concept of mobility in *Reconstructing mobility: Environmental*, *Behavioral*, *and Morphological Determinants* (eds Carlson, K. J. & Marchi, D.) 91–110 (Springer, 2014).

[16] Neustupný, E. *Prehistory of Bohemia 4*. *Eneolithic*. (The Institute of Archaeology of CAS, 2008).

[17] Price TD, Knipper C, Grupe G, Smrčka V (2004). Strontium isotopes and prehistoric human migration: The Bell Beaker period in Central. Europe. Eur. J. Archaeol.

[18] Sládek V, Berner M, Sailer R (2006). Mobility in Central European Late Eneolithic and Early Bronze Age: Tibial cross-sectional geometry. J. Archaeol. Sci..

[19] Sládek V, Berner M, Sailer R (2006). Mobility in Central European Late Eneolithic and Early Bronze Age: Femoral cross-sectional geometry. Am. J. Phys. Anthropol..

[20] Kolář J (2018). Population and forest dynamics during the Central European Eneolithic (4500–2000 BC). Archaeol. Anthropol. Sci..

[21] Jiráň, L. *Prehistory of Bohemia 5*. *The Bronze Age*. (The Institute of Archaeology of CAS, 2008).

[22] Kočár P, Dreslerová D (2010). Archaeobotanical finds of cultivated plants in the prehistory of the Czech Republic. Památky Archeologické.

[23] Venclová, N. *Prehistory of Bohemia 6. The Late Iron Age – The La Téne period*. (The Institute of Archaeology of CAS, 2008).

[24] Ruff C (2015). Gradual decline in mobility with the adoption of food production in Europe. Proc. Natl. Acad. Sci. USA.

[25] Poláček, L. Great Moravia, the power centre at Mikulčice and the issue of the socioeconomic structure. In *Studien zum Burgwall von Mikulčice VIII*. (eds Velemínský, P. & Poláček, L.) 11–44 (Archeological Institute of CAS, 2008).

[26] Molenda D (1976). Mining towns in Central-Eastern Europe in feudal times. Acta Pol.Hist.

[27] Horák J, Hejcman M (2013). Use of trace elements from historical mining for alluvial sediment dating. Soil & Water Res.

[28] Pachner, P. *Pohlavní rozdíly na lidské pánvi*. (Czech Academy of Science, 1937).

[29] Kujanová M, Bigoni L, Velemínská J, Velemínský P (2008). Limb bones asymmetry and stress in Medieval and recent populations of Central. Europe. Int. J. Osteoarchaeol.

[30] Bigoni L, Krajíček V, Sládek V, Velemínský P, Velemínská J (2013). Skull shape asymmetry and the socioeconomic structure of an Early Medieval central European society. Am. J. Phys. Anthrop..

[31] Bigoni L, Žaloudková M, Velemínská J, Velemínský P, Seichert V (2005). The occurrence of directional and fluctuating limb asymmetry in a recently identified collection of human bones. J. Natl. Mus. Nat. Hist. Ser.

[32] Stránská P, Likovský J, Velemínská J (2005). State of dental health and selected pathological conditions in the recent skulls from the Pachner’s collection. Slov. Antropol.

[33] Brůžek J (2002). A method for visual determination of sex, using the human hip bone. Am. J. Phys. Anthropol..

[34] Buikstra, J. E. & Ubelaker, H. D. Standards for data collection from human skeletal remains. *Proc. Sem. Field. Mus. Nat. Hist*. (Arkansas Archeological Survey Research Series, 1994).

[35] Schmitt A, Murail P, Cunha E, Rougé D (2002). Variability of the pattern of aging on the human skeleton: Evidence from bone indicators and implications on age at death estimation. J. Forensic Sci..

[36] Brzobohatá H, Prokop J, Horák M, Jančárek A, Velemínská J (2012). Accuracy and benefits of 3D bone surface modelling: a comparison of two methods of surface data acquisition reconstructed by laser scanning and computed tomography outputs. Coll. Antropol.

[37] Adams JW, Olah A, McCurry MR, Potze S (2015). Surface model and tomographic archive of fossil primate and other mammal holotype and paratype specimens of the Ditsong National Museum of Natural History, Pretoria, South Africa. Plos One.

[38] Bookstein FL (1997). Landmark methods for forms without landmarks: Morphometrics of group differences in outline shape. Med. Image Anal..

[39] Bookstein, F. L. *Morphometric Tools For Landmark Data: Geometry and Biology* (Cambridge University Press, 1991).

[40] von Cramon-Taubadel N, Frazier BC, Lahr MM (2007). The problem of assessing landmark error in geometric morphometrics: Theory, methods, and modifications. Am. J. Phys. Anthropol..

[41] De Groote I, Lockwood CA, Aiello LC (2010). Technical note: A new method for measuring long bone curvature using 3D landmarks and semilandmarks. Am. J. Phys. Anthropol..

[42] Biewener AA (1983). Allometry of quadrupedal locomotion: The scaling of duty factor, bone curvature and limb orientation to body size. J. Exp. Biol..

[43] Stern J, Jungers W, Susman R (1995). Quantifying phalangeal curvature - an empirical comparison of alternative methods. Am. J. Phys. Anthropol..

[44] Bruns W, Bruce M, Prescott G, Maffulli N (2002). Temporal trends in femoral curvature and length in Medieval and modern Scotland. Am. J. Phys. Anthropol..

[45] Deane AS, Kremer EP, Begun DR (2005). New approach to quantifying anatomical curvatures using highresolution polynomial curve fitting (HR-PCF). Am. J. Phys. Anthropol..

[46] Galtés I, Jordana X, Manyosa J, Malgosa A (2009). Functional implications of radial diaphyseal curvature. Am. J. Phys. Anthropol..

[47] Gunz P, Ramsier M, Kuhrig M, Hublin J-J, Spoor F (2012). The mammalian bony labyrinth reconsidered, introducing a comprehensive geometric morphometric approach. J. Anat.

[48] Botton-Divet L, Houssaye A, Herrel A, Fabre A-C, Cornette R (2015). Tools for quantitative form description: An evaluation of different software packages for semilandmark analysis. PeerJ.

[49] Rosenberger AL (2015). 1.32 ± 0.11 Ma age for underwater remains constrain antiquity and longevity of the Dominican primate Antillothrix bernensis. J. Hum. Evol..

[50] Wilson LAB, Humphrey LT (2017). Voyaging into the third dimension: A perspective on virtual methods and their application to studies of juvenile sex estimation and the ontogeny of sexual dimorphism. Forensic Sci. Int..

[51] De Groote I (2011). Femoral curvature in Neanderthals and modern humans: A 3D geometric morphometric analysis. J. Hum. Evol..

[52] Adams, D. C., Collyer, M. & Kaliontzopoulou, A. Geomorph: Geometric Morphometric Analyses of 2D/3D Landmark Data. R package version 3.0.6, https://CRAN.R-project.org/package=geomorph (2018).

[53] Dryden, I. L. & Mardia, K. V. *Statistical Shape Analysis*. (Wiley, 1998).

[54] Curran J (2017). Hotelling: Hotelling’s T2 Test and Variants. R package version.

[55] Peres-Neto PR, Jackson DA, Somers KM (2005). How many principal components? Stopping rules for determining the number of non-trivial axes revisited. Comput. Stat. Data Anal..

[56] Morphome 3cs 2.0 version. Department of Software and Computer Science Education, Faculty of Mathematics and Physics, Charles University, Prague, Czech Republic, http://www.morphome3cs.com.

[57] R Core Team. R: A language and environment for statistical computing. R Foundation for Statistical Computing, Vienna, Austria, http://www.R-project.org/ (2018).

[58] Ibrová A (2017). Facial skeleton asymmetry and its relationship to mastication in the Early Medieval period (Great Moravian Empire, Mikulčice, 9–10th century). Arch. Oral. Biol..

[59] Havelková P, Villotte S, Velemínský P, Poláček L, Dobisíková M (2011). Enthesopathies and activity patterns in the Early Medieval Great Moravian population: Evidence of division of labour. Int. J. Osteoarchaeol.

[60] Komar DA, Grivas C (2008). Manufactured populations: What do contemporary reference skeletal collections represent? A comparative study using the Maxwell Museum documented collection. Am. J. Phys. Anthropol..

[61] Ruff C (1987). Sexual dimorphism in human lower limb bone structure: Relationship to subsistence strategy and sexual division of labor. J. Hum. Evol..

[62] Brzobohatá H, Krajíček V, Horák Z, Velemínská J (2016). Sexual dimorphism of the human tibia through time: Insights into shape variation using a surface-based approach. Plos One.

[63] Brzobohatá H, Krajíček V, Velemínský P, Poláček L, Velemínská J (2014). The shape variability of human tibial epiphyses in an early medieval Great Moravian population (9th–10th century AD): A geometric morphometric assessment. Antropol. Anz.

[64] Brzobohatá H, Krajíček V, Horák Z, Sedlak P, Velemínská J (2016). Diachronic changes in size and shape of human proximal tibia in Central Europe during the latest 1200 years. Homo.

[65] Ruff CB, Hayes WC (1988). Sex differences in age-related remodeling of the femur and tibia. J. Orthop. Res..

[66] Ruff, C. Biomechanical analysis of archaeological human skeletons. In *Biological Anthropology of The Human Skeleton* (eds Katzenberg, M. & Saunders, S.) 71–102 (Wiley-Liss, 2000).

[67] Sparacello, V. S., Marchi, D. & Shaw, C. N. The importance of considering fibular robusticity when inferring the mobility patterns of past populations. In *Reconstructing Mobility: Environmental, Behavioral and Morphological Determinants* (eds Carlson, K. J. & Marchi, D.) 91–110 (Springer, 2014).

[68] Svoboda, J., Vašků, Z. & Cílek, V. *Velká kniha o klimatu zemí Koruny české*. (Regia, 2003).

[69] Wallace IJ, Tommasini SM, Judex S, Garland T, Demes B (2012). Genetic variations and physical activity as determinants of limb bone morphology: An experimental approach using a mouse model. Am. J. Phys. Anthropol..

[70] von Cramon-Taubadel N, Stock JT, Pinhasi R (2013). Skull and limb morphology differentially track population history and environmental factors in the transition to agriculture in Europe. Proc. Biol. Sci.

[71] Auerbach BM, Gooding AF, Shaw CN, Sylvester AD (2017). The relative position of the human fibula to the tibia influences cross-sectional properties of the tibia. Am. J. Phys. Anthropol..

[72] Ruff CB, Hayes WC (1984). Age changes in geometry and mineral content of the lower limbs. An. Biomed. Eng.

[73] Ding M, Odgaard A, Linde F, Hvid I (2002). Age-related variations in the microstructure of human tibial cancellous bone. J. Orthop. Res..

[74] Ural A, Vashishth D (2006). Interactions between microstructural and geometrical adaptation in human cortical bone. J. Orthop. Res..

[75] Stevens SD, Viðarsdóttir US (2008). Morphological changes in the shape of the non-pathological bony knee joint with age: A morphometric analysis of the distal femur and proximal tibia in three populations of known age at death. Int. J. Osteoarchaeol.

