# Supplementary material for: Three-dimensional geometry of human tibial anterior curvature in chronologically distinct population samples of Central Europeans (2900 BC – 21st century AD)
Source: Sci Rep. 2019 Mar 12;9:4234. doi: 10.1038/s41598-019-40625-3 (PMC6414627; doi:10.1038/s41598-019-40625-3)
Supplement: Supplementary file 1 — Three-dimensional geometry of human tibial anterior curvature in chronologically distinct population samples of Central Europeans (2900 BC – 21st century AD) [file 41598_2019_40625_MOESM1_ESM.pdf]

SUPPLEMENTARY INFORMATION

Three-dimensional geometry of human tibial anterior curvature in chronologically distinct population samples of Central Europeans (2900 BC – 21st century AD)

Hana Brzobohatá, Václav Krajíček, Petr Velemínský & Jana Velemínská

Supplementary Information includes Supplementary Table S1-S2 and Supplementary Figures S1-S5.

Supplementary Table S1 Summary of p-values indicating the statistical significance of diachronic sex group differences in 3D anterior tibial crest curvature, assessed using Hotelling's T2 test with 10,000 permutations (significance level of $p < 0.05$, displayed in bold) (abbreviations as in Fig. 2).

**MALES**						
	**ENEOL**	**BRONZE**	**IRON**	**EMED**	**LMED**	**20th CENT.**
**BRONZE**	0.673					
**IRON**	0.083	**0.028**				
**EMED**	**<0.001**	**<0.001**	**0.001**			
**LMED**	0.096	**0.002**	**<0.001**	**0.036**		
**20th CENT.**	**<0.001**	**<0.001**	**<0.001**	**<0.001**	**<0.001**	
**21st CENT.**	**<0.001**	**<0.001**	**<0.001**	**<0.001**	**<0.001**	0.750
**FEMALES**						
	**ENEOL**	**BRONZE**	**IRON**	**EMED**	**LMED**	**20th CENT.**
**BRONZE**	0.887					
**IRON**	0.270	0.103				
**EMED**	**0.005**	**<0.001**	0.055			
**LMED**	0.263	**0.016**	0.226	0.817		
**20th CENT.**	**<0.001**	**<0.001**	**<0.001**	**0.010**	0.168	
**21st CENT.**	**<0.001**	**<0.001**	**<0.001**	**<0.001**	**0.007**	0.065

Supplementary Table S2 Results of ANOVA performed on curve length regressed against shape variables showing no significant interaction between shape and size in sex-groups of diachronic samples (N, number; M, males; F, females).

Dataset	Sex	N of significant components	Explained variance (%)	p-value
**ENEOL**	M	4	70.01	0.620
	F	2	67.92	0.051
**BRONZE**	M	4	76.74	0.367
	F	5	79.71	0.624
**IRON**	M	3	68.28	0.217
	F	2	60.82	0.580
**EMED**	M	4	77.74	0.267
	F	6	79.80	0.912
**LMED**	M	3	73.39	0.200
	F	2	63.15	0.208
**20th CENT.**	M	4	77.41	0.241
	F	3	73.80	0.918
**21st CENT.**	M	2	71.01	0.580
	F	3	74.48	0.927

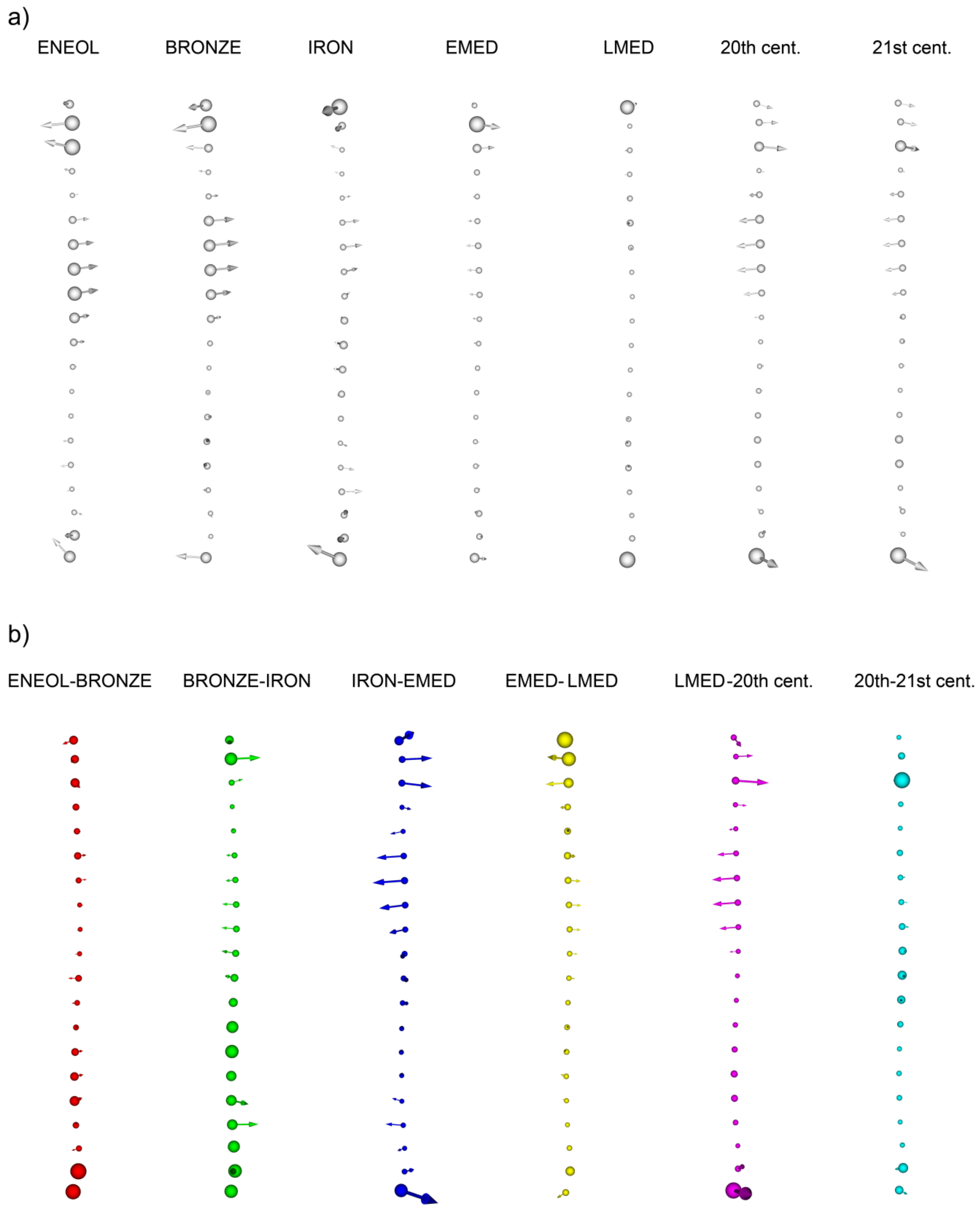

Supplementary Figure S1. **(a)** Shape differences in particular male samples with respect to chronological age. Medial view of anterior tibial curvature, with arrows showing the A-P shape change from the mean curve of pooled sample toward the mean curve of the chronologically specified dataset. **(b)** Vector plot showing the shape differences in the left anterior tibial A-P curvature between chronologically adjacent groups. Chronologically older diachronic groups are represented by circles and chronologically younger samples by arrow points (abbreviations as in Fig. 2).

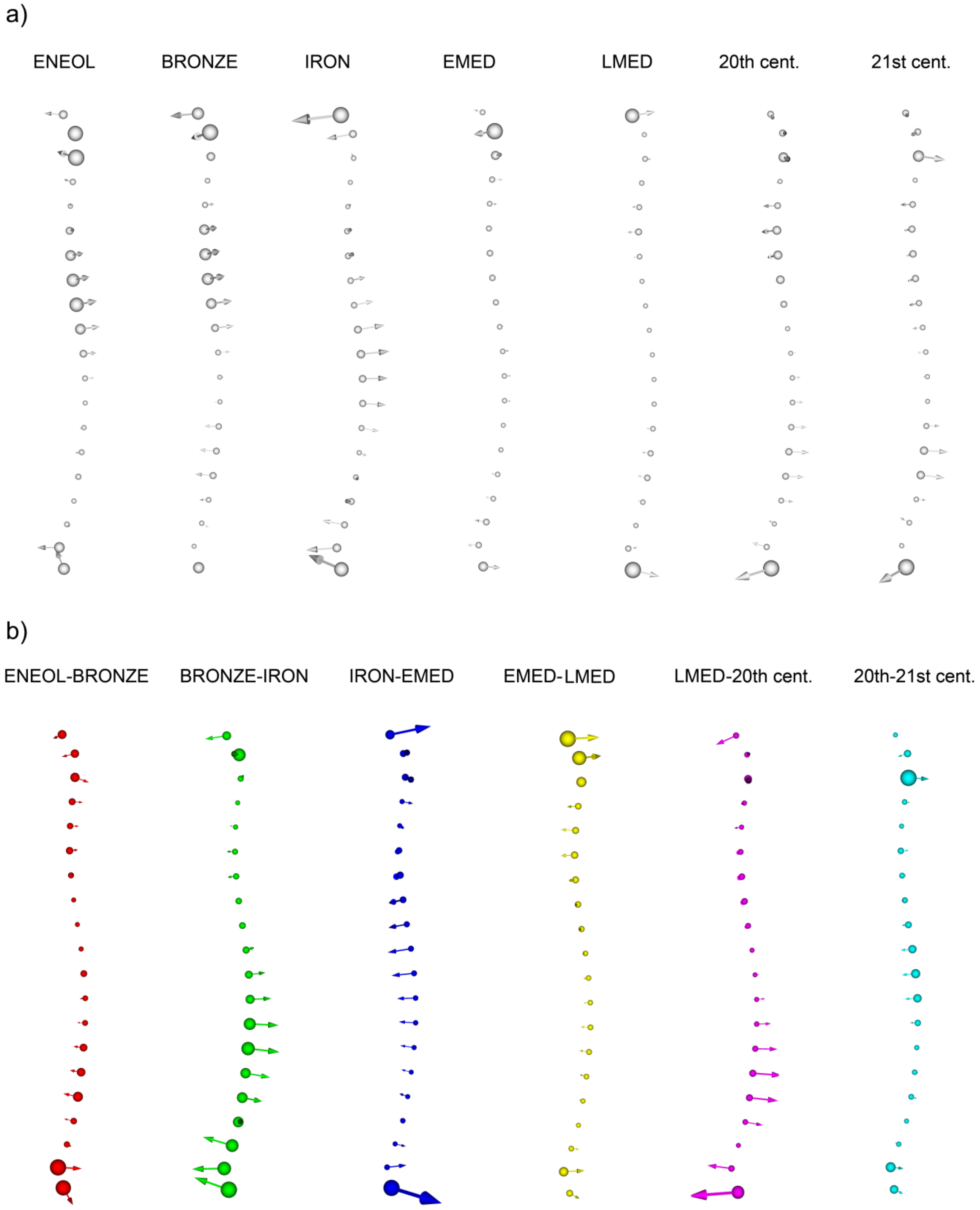

Supplementary Figure S2. **(a)** Shape differences in particular male samples with respect to chronological age. Anterior view of anterior tibial curvature, with arrows showing the M-L shape change from the mean curve of pooled sample toward the mean curve of the chronologically specified dataset. **(b)** Vector plot showing the shape differences in the left anterior tibial M-L curvature between chronologically adjacent groups. Chronologically older diachronic groups are represented by circles and chronologically younger samples by arrow points (abbreviations as in Fig.2).

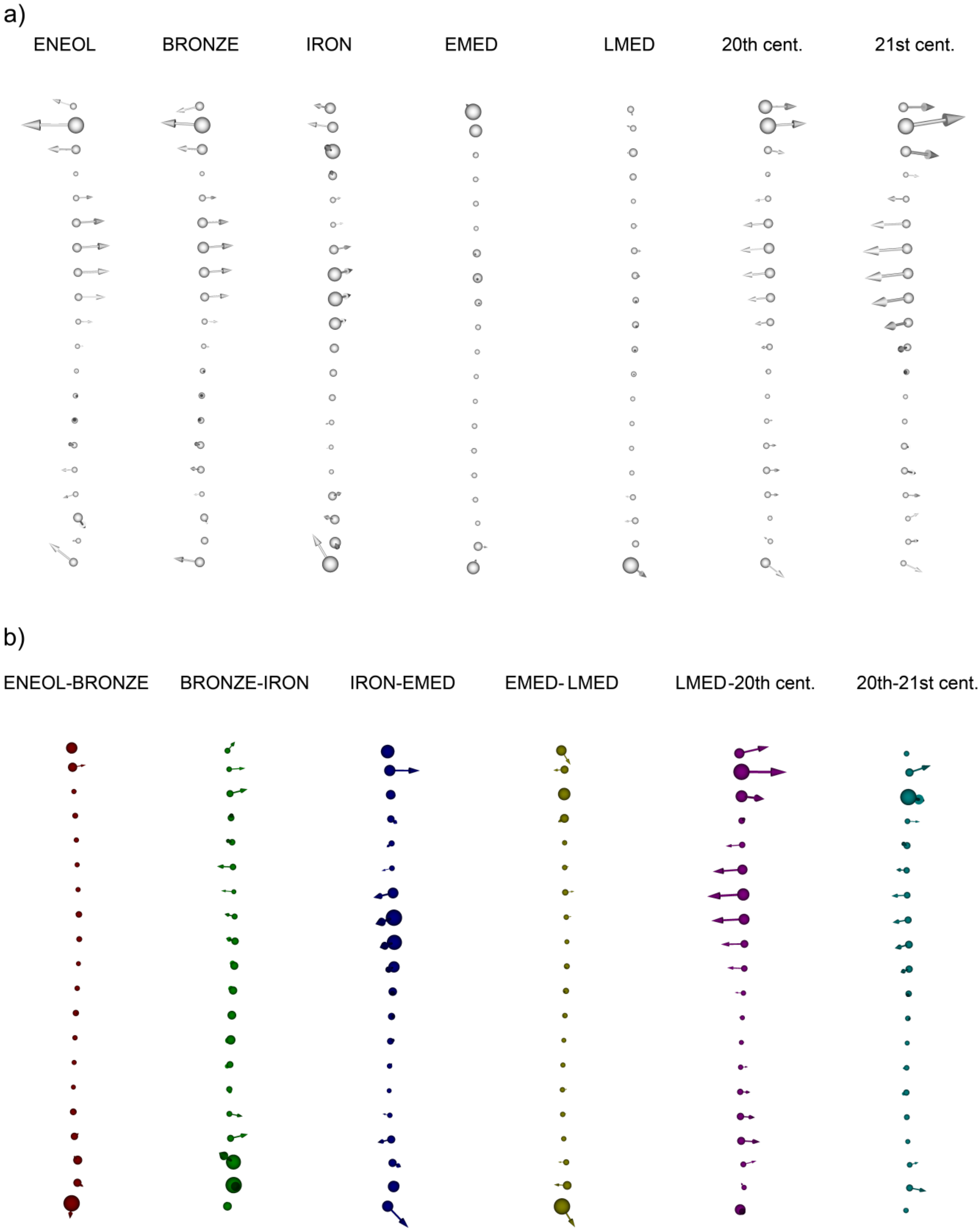

Supplementary Figure S3. **(a)** Shape differences in particular female samples with respect to chronological age. Medial view of anterior tibial curvature, with arrows showing the A-P shape change from the mean curve of pooled sample toward the mean curve of the chronologically specified dataset. **(b)** Vector plot showing the shape differences in the left anterior tibial A-P curvature between chronologically adjacent groups. Chronologically older diachronic groups are represented by circles and chronologically younger samples by arrow points (abbreviations as in Fig.2).

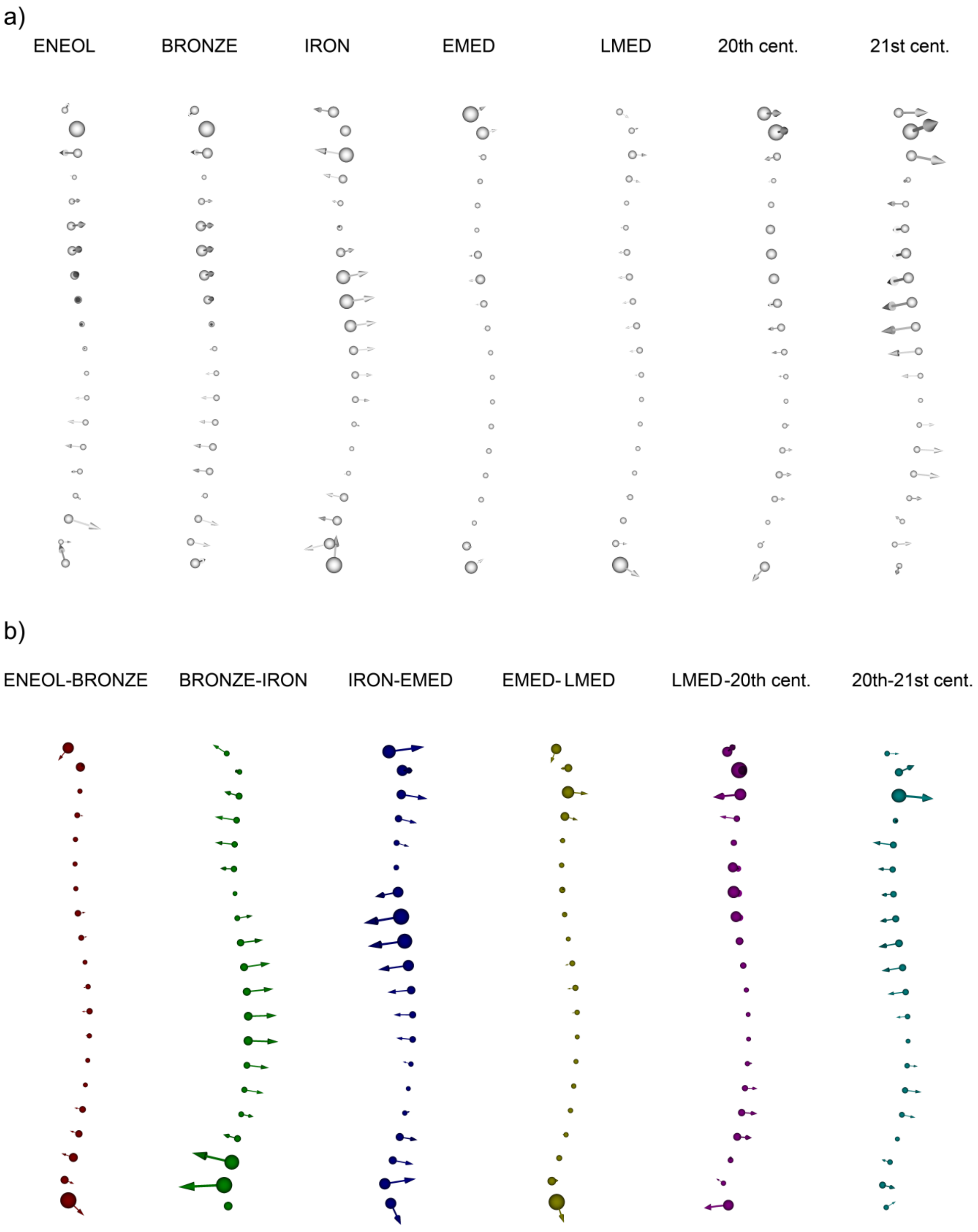

Supplementary Figure S4. **(a)** Shape differences in particular female samples with respect to chronological age. Anterior view of anterior tibial curvature, with arrows showing the M-L shape change from the mean curve of pooled sample toward the mean curve of the chronologically specified dataset. **(b)** Vector plot showing the shape differences in the left anterior tibial M-L curvature between chronologically adjacent groups. Chronologically older diachronic groups are represented by circles and chronologically younger samples by arrow points (abbreviations as in Fig.2).

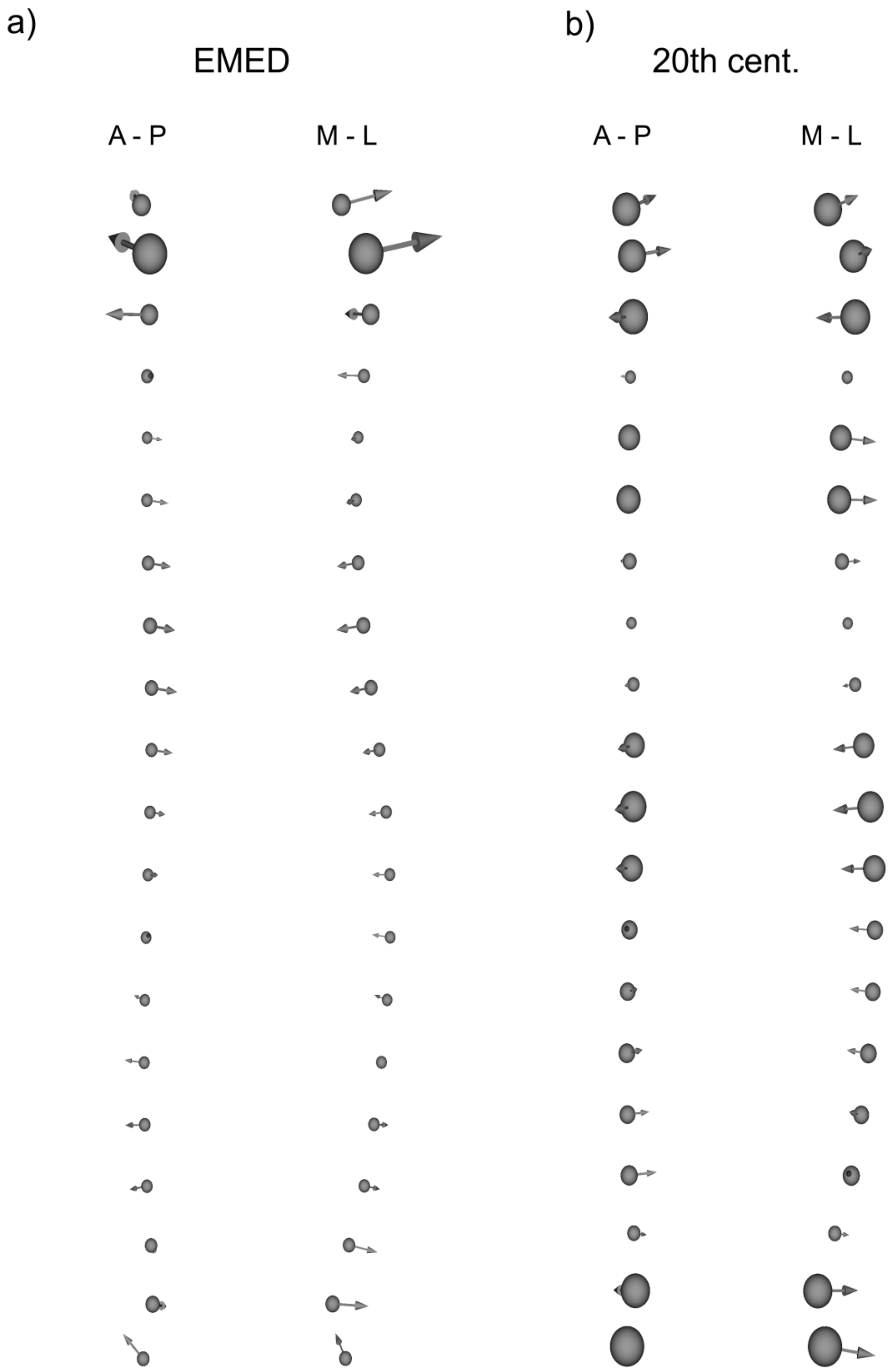

Supplementary Figure S5. Sex-based shape differences in anterior tibial curvature. Vector plots showing the shape differences between males and females in the Early Medieval **(a)** and the 20th century **(b)** sample. Arrows indicate the direction of change from male to female tibiae (abbreviations as in Fig.2).
